# Regulatory Influence of Galanin and GALR1/GALR2 Receptors on Inflamed Uterus Contractility in Pigs

**DOI:** 10.3390/ijms22126415

**Published:** 2021-06-15

**Authors:** Barbara Jana, Jarosław Całka, Bartosz Miciński

**Affiliations:** 1 Division of Reproductive Biology, Institute of Animal Reproduction and Food Research of the Polish Academy of Sciences, Tuwima 10, 10-748 Olsztyn, Poland; 2 Department of Clinical Physiology, Faculty of Veterinary Medicine, University of Warmia and Mazury, Oczapowskiego 14, 11-041 Olsztyn, Poland; calkaj@uwm.edu.pl (J.C.); bartosz.micinski@uwm.edu.pl (B.M.)

**Keywords:** uterus, inflammation, contractility, galanin, galanin receptor, pig

## Abstract

Uterine inflammation is a very common and serious pathology in domestic animals, the development and progression of which often result from disturbed myometrial contractility. We investigated the effect of inflammation on the protein expression of galanin (GAL) receptor subtypes (GALR)1 and GALR2 in myometrium and their role in the contractile amplitude and frequency of an inflamed gilt uterus. The gilts of the *E. coli* and SAL groups received *E. coli* suspension or saline in their uteri, respectively, and only laparotomy was performed (CON group). Eight days later, the *E. coli* group developed severe acute endometritis and lowered GALR1 protein expression in the myometrium. Compared to the pretreatment period, GAL (10^−7^ M) reduced the amplitude and frequency in myometrium and endometrium/myometrium of the CON and SAL groups, the amplitude in both stripes and frequency in endometrium/myometrium of the *E. coli* group. In this group, myometrial frequency after using GAL increased, and it was higher than in other groups. GALR2 antagonist diminished the decrease in amplitude in myometrium and the frequency in endometrium/myometrium (SAL, *E. coli* groups) induced by GAL (10^−7^ M). GALR1/GALR2 antagonist and GAL (10^−7^ M) reversed the decrease in amplitude and diminished the decrease in frequency in both examined stripes (CON, SAL groups), and diminished the drop in amplitude and abolished the rise in the frequency in the myometrium (*E. coli* group). In summary, the inflammation reduced GALR1 protein expression in pig myometrium, and GALR1 and GALR2 participated in the contractile regulation of an inflamed uterus.

## 1. Introduction

Uterine inflammation is a common reproductive disorder occurring in domestic animals, as well as in women. In pigs, endometritis and metritis are a cause of reduced profitability in animal production [1]. These pathologies are predominantly evoked by bacterial organisms and take place mainly in the postpartum period. To develop inflammation, impairment of myometrial contractile activity and/or immunological response is significant [2,3]. Difficult labor and fetal membrane retention contribute to an expansion of this disorder [4,5]. In more advanced cases of inflammation, the uterus is filled with mucopurulent exudate, and the myometrium loses contractility [6,7]. Until now, the factors influencing the contractility of the uterus with an inflammatory state have been poorly understood. In the inflamed uterus, a rise of the production and secretion of prostaglandin (PG)F_2_α, PGE_2_, and leukotrienes (LT)B_4_ and LTC_4_ in cows [2,8] and pigs [9,10], as well as PGI_2_ [11] in pigs, was noted. These PGs [11,12,13] and LTC_4_ and LTD_4_ [14] significantly affect the contractility of the porcine inflamed uterus. In postpartum cows, oxytocin positively influences the inflamed uterus contractility, while PGF_2α_ induces an initial decrease in contraction, followed by an increase [15]. In reference to the regulation of uterine contractility with inflammation by neurotransmitters, the role of noradrenaline (NA), acetylcholine (ACh), neuropeptide Y (NPY), somatostatin (SOM), and vasoactive intestinal peptide (VIP) [11,13,16,17,18,19] and the expression of adrenergic and muscarinic receptors [20,21] in diseased porcine organs were reported. Moreover, in the pregnant rats with uterine inflammation, tumor necrosis factor-α mediates the increase of uterine relaxation induced by β_2_-adrenergic receptor agonists [22].

Galanin (GAL), GAL-like peptide, alarin, and GAL-message-associated peptide belong to the GAL family. GAL is a principal member of this family, and its biological action is exerted through interaction with the following kinds of receptors: GALR1, GALR2, and GALR3. All these receptors are G-protein-coupled receptors, and their expression is often found in both the central and peripheral nervous systems and peripheral organs. GAL and its receptors have been implicated in many diverse functions, including stress and inflammation [23,24].

In pigs, GAL has been found in the neurons of the inferior mesenteric ganglion (CaMG) [25] and dorsal root ganglia (DRGs) [26] projecting to the uterus. GAL-positive nerve fibers are situated in the rat [27] and porcine [28] endometrium and myometrium. In the myometrium, GAL-immunoreactive fibers are present among muscle cells and around blood vessels. In rats, the GALR2 mRNA is present in the myometrium [29], and GAL stimulates uterine contractility [27,29] under physiological conditions. In turn, other studies on rats and perimenopausal-aged women have not found a significant GAL effect on uterine contractility [30,31].

With regard to the innervation of an inflamed uterus, it has been shown that in the DRGs, endometritis increased the population of perikarya-expressing substance P (SP) in rats [32] and sets of SP- and GAL-positive uterine perikarya in gilts [26], suggesting a role of these neuropeptides in the sensory stimuli transmission from pathologically changed organs to the spinal cord, as well as in antidromic modulation of uterus function. In the DRGs of gilts suffering from endometritis, the numbers of uterine perikarya immunopositive to NA, NPY, GAL, or vasoactive intestinal peptide were increased [33]. It was hypothesized that the inflammatory process leads to alterations in GALR expression, and determines the participation of GAL in contractility of the uterus with inflammation. Knowledge of the receptor bases of the GAL effect may allow better recognition of the mechanism of neurogenic regulation of the inflamed uterus function. Finally, identification and functional characterization of GALRs in the inflamed uterus’ contractility can improve the prevention and treatment of uterine inflammation in domestic animals. It is also worth adding that GALR1 and GALR2 expression levels in the gastrointestinal tract are high, while GALR3 expression level is low [34].

Thus, we studied (1) the effect of inflammation on GALR1 and GALR2 protein expression in myometrium, and (2) the role of these receptors in GAL-influenced contractile amplitude and frequency of inflamed gilt uterus. Importantly, the results of these studies, performed on the domestic pig model (with high similarity of anatomical structures and physiological processes to human), can be extrapolated to human medicine, allowing a better understanding of the mechanisms associated with uterine inflammation [35].

## 2. Results

### 2.1. Expression of GALR1 and GALR2 Proteins

**Figure 1:**
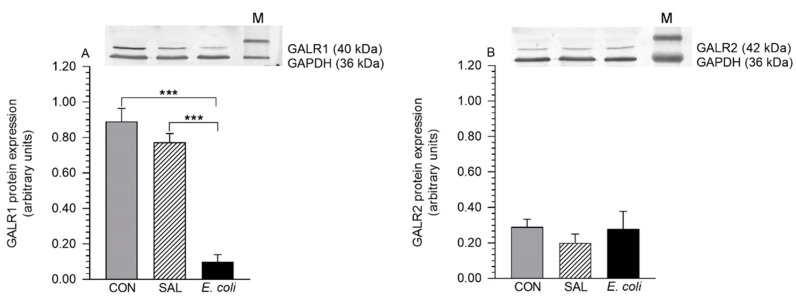
The protein expression of GALR1 (**A**) and GALR2 (**B**) in the myometrial layer of gilts from the control (CON), saline (SAL), and *E. coli* groups, estimated by Western blot analysis. Representative blots are shown in the top panels. Protein levels of studied receptors are expressed as the mean ± SEM of ratios of glyceraldehyde-3-phosphate dehydrogenase (GAPDH). *** *p* < 0.001 compared between groups for the same type of receptor. M—marker.

Protein bands indicated the molecular weights: 40 kDa for GALR1 and 42 kDa for GALR2 (Figure 1A,B). Western blot analysis of the pig duodenum (positive control) revealed bands at 40 and 42 kDa, and they were recognized as GALR1 and GALR2 proteins, respectively (Supplementary Figure S1). The bands were not visible after omitting primary antibodies (data not shown).

The myometrial GALR1 protein expression in the inflammation-affected uteri was reduced (*p* < 0.001) compared to the control and saline-injected organs (Figure 1A). The GALR2 protein contents in the myometrial tissues of all studied groups did not differ significantly (Figure 1B).

### 2.2. Distribution of GALR1 and GALR2

**Figure 2:**
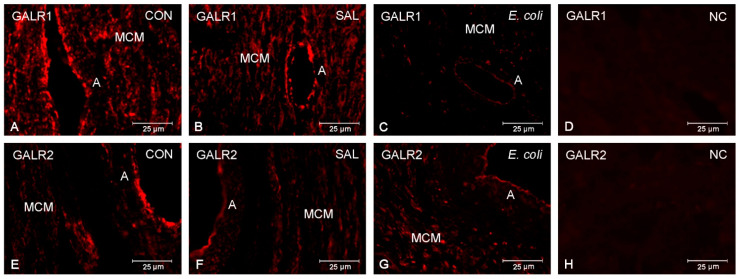
The pictures present GALR1 (**A**–**C**) and GALR2 (**E**–**G**) in the myometrial layer of gilts from the control (CON), saline (SAL), and *E. coli* groups. Positive immunoreaction to GALR1 was found in muscle cells, as well as in arteries (endothelium, muscle layer) of myometrium, of the control (**A**), saline-injected (**B**), and inflammatory-changed (**C**) uteri. Similarly, GALR2 was expressed in these structures in the control (**E**), saline-injected (**F**), and inflamed (**G**) organs. Negative control (NC) for GALR1 (**D**) and GALR2 (**H**) was obtained following the use of normal rabbit IgG instead of the primary antibodies. MCM—myometrial muscle cells; A—artery.

Immunofluorescence revealed GALR1 and GALR2 occurrence in the porcine duodenum (Supplementary Figure S2). No labeling of these receptors was detected after using normal rabbit IgG instead of the primary antibodies (Figure 2D,H). The expression of GALR1 and GALR2 was found in the myometrial muscle cells, as well as in endothelium and muscle layer of blood vessels, in the control (Figure 2A,E), saline-injected (Figure 2B,F), and inflamed (Figure 2C,G) uteri.

### 2.3. GAL Influence on the Myometrium Contractility

#### 2.3.1. Comparison of the GAL Influence to the Period before Its Application

**Figure 3:**
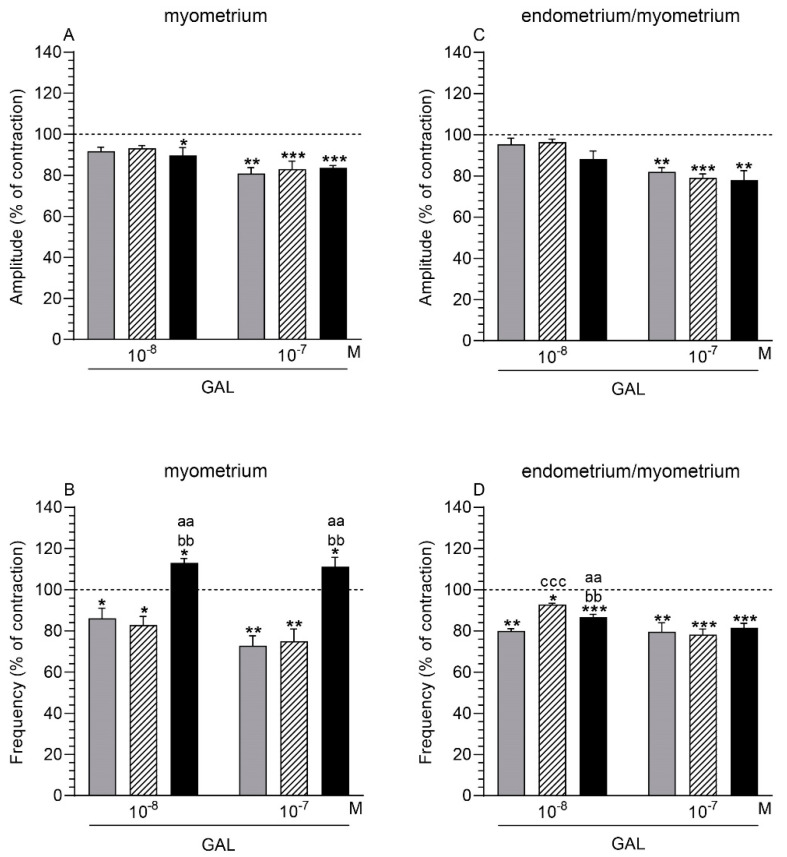
The influence of galanin (GAL) on the contractile amplitude (**A**,**C**) and frequency (**B**,**D**) in the myometrium (**A**,**B**) and endometrium/myometrium (**C**,**D**) stripes of gilts from the CON (grey bars), SAL (hatched bars), and *E. coli* (black bars) groups. Results were calculated for five gilts in each group. The effects of individual GAL doses are depicted as percentages (mean ± SEM) of the baseline (pretreatment period) contractile amplitude and frequency, taken as 100% (horizontal lines). * *p* < 0.05, ** *p* < 0.01, *** *p* < 0.001 compared to the basal value in each group; aa *p* < 0.01 compared between the CON and *E. coli* groups for the same treatment; bb *p* < 0.01 compared between the SAL and *E. coli* groups for the same treatment; ccc *p* < 0.001 compared between the CON and SAL groups for the same treatment.

After using GAL (10^−7^ M), the amplitude in the myometrium was significantly lowered in the control, saline-injected, and inflamed uteri (Figure 3A). In the inflamed uteri, a similar reaction was also evoked by GAL at a dose of 10^−8^ M. GAL (10^−8^, 10^−7^ M) significantly decreased the frequency in the control and saline-injected organs, while significantly increasing this parameter in the inflamed uteri (Figure 3B).

#### 2.3.2. Comparison of the GAL Influence between Groups

The myometrial frequency in the inflamed uteri was significantly enhanced after using GAL (10^−8^, 10^−7^ M) vs. other groups (Figure 3B). In all groups, the amplitude in the myometrium did not differ significantly after the application of GAL (10^−8^, 10^−7^ M) (Figure 3A).

### 2.4. GAL Influence on the Endometrium/Myometrium Contractility

#### 2.4.1. Comparison of the GAL Influence to the Period before its Application

In the control, saline-injected, and inflamed uteri, GAL (10^−7^ M) significantly decreased the amplitude in the endometrium/myometrium (Figure 3C). GAL (10^−8^, 10^−7^ M) significantly reduced the frequency in the tissues of all groups (Figure 3D).

#### 2.4.2. Comparison of the GAL Influence between Groups

In response to GAL (10^−8^ M), the frequency in the endometrium/myometrium of the inflamed uteri significantly increased compared to the control uteri, and significantly decreased vs the saline-injected uteri (Figure 3D). Moreover, in the endometrium/myometrium of these organs, the frequency was significantly higher than in the control uteri. The amplitude in the endometrium/myometrium of all groups did not differ significantly in response to GAL (10^−8^, 10^−7^ M) (Figure 3C).

### 2.5. GALR2 Antagonist and GAL Influence on the Myometrium Contractility

#### 2.5.1. Comparison of the Antagonist and GAL Influence to the Period before Their Application

**Figure 4:**
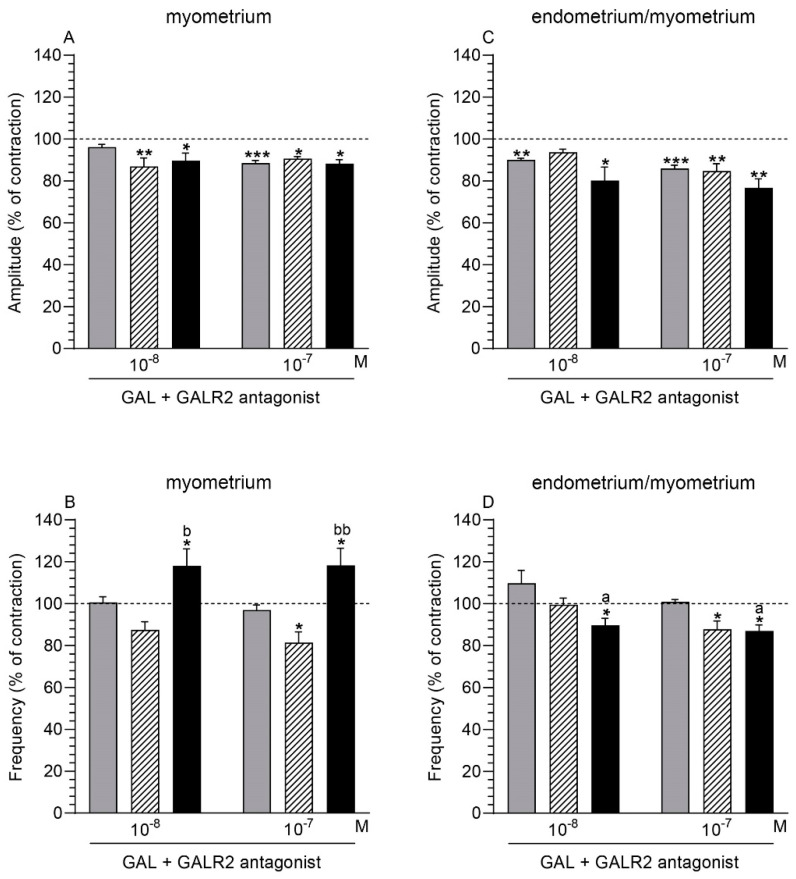
The influence of galanin (GAL) on the contractile amplitude (**A**,**C**) and frequency (**B**,**D**) in the myometrium (**A**,**B**) and endometrium/myometrium (**C**,**D**) stripes of gilts from the CON (grey bars), SAL (hatched bars), and *E. coli* (black bars) groups after using GALR2 antagonist (at a dose of 10^−6^ M). Results were calculated for five gilts in each group. The effects of antagonist and individual GAL doses are depicted as percentages (mean ± SEM) of the baseline (pretreatment period) contractile amplitude and frequency, taken as 100% (horizontal lines). * *p* < 0.05, ** *p* < 0.01, *** *p* < 0.001 compared to the basal value in each group; a *p* < 0.05 compared between the CON and *E. coli* groups for the same treatment; b *p* < 0.05, bb *p* < 0.01 compared between the SAL and *E. coli* groups for the same treatment.

Following the application of GALR2 antagonist (10^−6^ M) with GAL (10^−8^, 10^−7^ M), the myometrial amplitude of the saline-injected and inflamed uteri was significantly lowered (Figure 4A). A similar finding was also found in the myometrium of the control organs after administration of GALR2 antagonist (10^−6^ M) together with GAL at a dose of 10^−7^ M. GALR2 antagonist (10^−6^ M) and GAL (10^−7^ M) significantly decreased the myometrial frequency in the saline-injected uteri (Figure 4B). GALR2 antagonist (10^−6^ M) and GAL (10^−8^, 10^−7^ M) significantly augmented this parameter in the myometrium of the inflamed organs.

#### 2.5.2. Comparison of the Antagonist and GAL Influence between Groups

The myometrial frequency in the inflamed uteri under the influence of GALR2 antagonist (10^−6^ M) with GAL (10^−8^, 10^−7^ M) was significantly increased vs. the saline-injected organs (Figure 4B). After using GALR2 antagonist (10^−6^ M) and GAL (10^−8^, 10^−7^ M), the amplitude in the myometrium did not differ significantly between all groups (Figure 4A).

### 2.6. GALR2 Antagonist and GAL Influence on the Endometrium/Myometrium Contractility

#### 2.6.1. Comparison of the Antagonist and GAL Influence to the Period before Their Application

GALR2 antagonist (10^−6^ M) and GAL (10^−8^, 10^−7^ M) significantly decreased the amplitude in the endometrium/myometrium of the control and inflamed uteri (Figure 4C). In the saline-injected organs, a significant drop was revealed in the endometrium/myometrium following the application of GALR2 antagonist (10^−6^ M) with GAL at a dose of 10^−7^ M. In these uteri, GALR2 antagonist (10^−6^ M) together with GAL (10^−7^ M) significantly decreased the frequency in the endometrium/myometrium (Figure 4D). In the endometrium/myometrium of the inflamed organs, GALR2 antagonist (10^−6^ M) with GAL (10^−8^, 10^−7^ M) caused a significant drop in the frequency.

#### 2.6.2. Comparison of the Antagonist and GAL Influence between Groups

GALR2 antagonist (10^−6^ M) with GAL (10^−8^, 10^−7^ M) led to a significant reduction in the frequency in the endometrium/myometrium of the inflamed uteri vs. the control organs (Figure 4D). After the application of GALR2 antagonist (10^−6^ M) together with GAL (10^−8^, 10^−7^ M), the amplitude in the endometrium/myometrium did not differ significantly between all groups (Figure 4C).

### 2.7. GALR1/GALR2 Antagonist and GAL Influence on the Myometrium Contractility

#### 2.7.1. Comparison of the Antagonist and GAL Influence to the Period before Their Application

**Figure 5:**
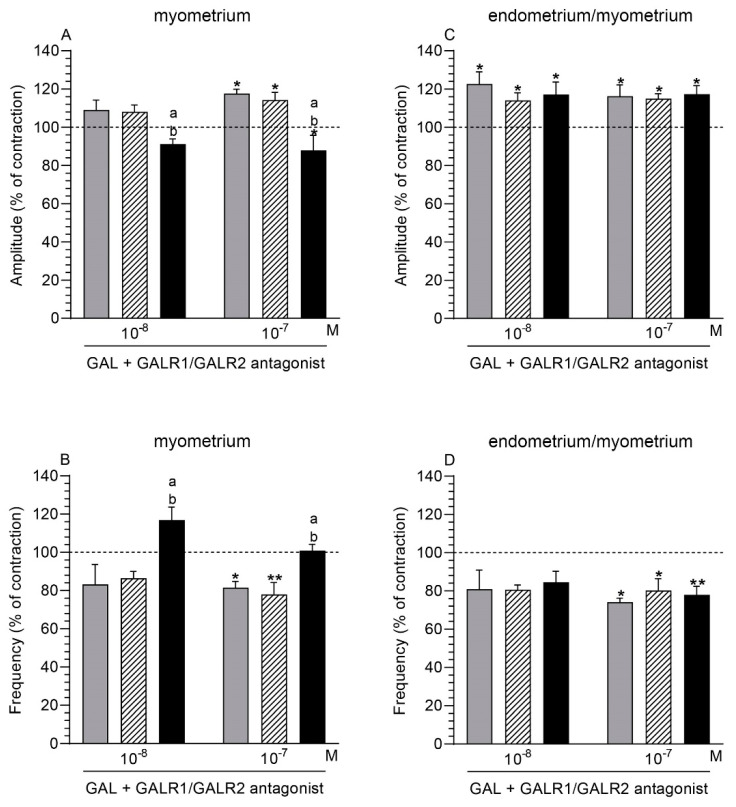
The influence of galanin (GAL) on the contractile amplitude (**A**,**C**) and frequency (**B**,**D**) in the myometrium (**A**,**B**) and endometrium/myometrium (**C**,**D**) stripes of gilts from the CON (grey bars), SAL (hatched bars), and *E. coli* (black bars) groups after using GALR1/GALR2 antagonist (at a dose of 10^−6^ M). Results were calculated for five gilts in each group. The effects of antagonist and individual GAL doses are depicted as percentages (mean ± SEM) of the baseline (pretreatment period) contractile amplitude and frequency, taken as 100% (horizontal lines). * *p* < 0.05, ** *p* < 0.01 compared to the basal value in each group; a *p* < 0.05 compared between the CON and *E. coli* groups for the same treatment; b *p* < 0.05 compared between the SAL and *E. coli* groups for the same treatment.

After using GALR1/GALR2 antagonist (10^−6^ M) and GAL (10^−7^ M), the myometrial amplitude in the control and saline-injected uteri significantly increased, while it significantly decreased in the inflamed uteri (Figure 5A). Following treatment with GALR1/GALR2 antagonist (10^−6^ M) and GAL (10^−7^ M) in the myometrium of the control and saline-injected organs, a significant drop in the frequency was found (Figure 5B).

#### 2.7.2. Comparison of the Antagonist and GAL Influence between Groups

In the presence of GALR1/GALR2 antagonist (10^−6^ M) and GAL (10^−8^, 10^−7^ M), the myometrial amplitude in the inflamed uteri was significantly lower vs. the control and saline-injected organs (Figure 5A). After the addition of this antagonist (10^−6^ M) and GAL (10^−8^, 10^−7^ M), the frequency in the myometrium of the inflamed uteri was significantly enhanced compared to other groups (Figure 5B).

### 2.8. GALR1/GALR2 Antagonist and GAL Influence on the Endometrium/Myometrium Contractility

#### 2.8.1. Comparison of the Antagonist and GAL Influence to the Period before Their Application

GALR1/GALR2 antagonist (10^−6^ M) and GAL (10^−8^, 10^−7^ M) led to a significant rise of the amplitude in the endometrium/myometrium of all groups (Figure 5C). The antagonist (10^−6^ M) and GAL (10^−7^ M) significantly decreased the frequency in the tissues of the control, saline-injected, and inflamed uteri (Figure 5D).

#### 2.8.2. Comparison of the Antagonist and GAL Influence between Groups

In response to GALR1/GALR2 antagonist (10^−6^ M) and GAL (10^−8^, 10^−7^ M), the amplitude (Figure 5C) and frequency (Figure 5D) in the endometrium/myometrium of all groups did not differ significantly.

## 3. Discussion

The current report presents the action of endometritis on the GALR1 and GALR2 protein expression in the myometrium, and the importance of GAL, GALR1, and GALR2 in the contractile activity of a pig uterus with inflammation. The macroscopic and histopathologic examinations of uteri used in the present study were reported earlier [20]. Briefly, after *E. coli* injections, inflammatory exudate was present inside the uterine horns. The endometrium was red and swollen, with distinctly visible blood vessels. Histopathologically, a severe form of acute endometritis in these uteri was diagnosed by a significant increase in the number of neutrophils and luminal and/or glandular epithelium damage.

As mentioned earlier, GALRs are commonly found in the nervous system and the peripheral organs. For example, GALR1 and GALR2 mRNA expression in the gastrointestinal tract is high, while GALR3 mRNA expression is low [34]. To date, regarding the uterus, only the expression of GALR2 mRNA was found in the rat myometrium under physiological conditions [29]. In turn, the current research revealed for the first time both GALR1 and GALR2 protein expression in the myometrial tissues of healthy and inflamed uteri. The GALR1 and GALR2 protein expression was similar in the CON and SAL groups, which allows us to suppose that the intrauterine infusion of saline did not significantly affect the expression of these receptors. In relation to the CON and SAL groups, following intrauterine bacteria administration, a reduction of the GALR1 protein content and a lack of significant alteration in the GALR2 protein content in the myometrium were revealed. In turn, in mice epithelial cells lining the colon in response to *E. coli* [36], and in the colonic epithelial cells of mice administered dextran sulfate sodium [37], a transition rise in GALR1 protein expression was found. The discrepancies in the protein expression of GALR1 between the inflamed uterus and colon may be due to the tissue types and the time of their collection for the study after the application of inflammation-provoking factors. Moreover, the peripheral inflammation in the rat DRGs neurons led to a transient reduction in GALR1 mRNA expression [38] and a transient increase in GALR2 mRNA expression [39]. Although the GALR1 and GALR2 mRNA levels were not determined in the current study, we supposed that post-transcriptional modulation of GALRs may lead to low or unchanged expression of proteins. This was suggested in the study of the expression of interleukin-6 in the porcine endometrium [40]. The changed myometrial expression of GALR1 might be regulated by lipopolysaccharide originated from bacteria [2] and/or pro- and anti-inflammatory cytokines produced in great amounts [6,41] in the uterus with inflammation. It was presented that in the *E. coli*-infected murine colonic epithelial cells, the GALR1 expression is regulated via the nuclear factor-κB-mediated process [42].

It was also found that the inflammatory state did not affect the myometrial distribution of both GALR1- and GALR2- immunoreactivity. The expression of GALR1 and GALR2 in the muscle cells and blood-vessel cells in gilts from the three examined groups may indicate that these cells become targets for GAL in healthy and inflamed uteri.

The changes in the myometrial protein expression of GALR1 in uteri with inflammation and the positive immunoreaction for GALR1 and GALR2 in myometrial cells suggest the participation of these receptors in the myometrium activity of the inflamed uteri. The presence of GALR1 and GALR2 in all the examined groups’ blood vessels suggests the role of these receptors in the modulating effect of GAL on blood-vessel activity in the myometrium. It is known that GAL can reduce the cutaneous blood flow and inhibit inflammatory edema creation by GALR2 [43], and that GAL also indirectly exerts this effect, by stopping SP and calcitonin gene-related peptide release [44]. In addition, GAL plays a role in central cardiovascular regulation [45].

The importance of the galinergic system in contractility of the uterus with inflammation was also studied. This is due to the high importance of disturbances of this uterine activity for the origin and control of inflammation and consequences of this process [2,6]. It is known that the effect of PGs, LTs, NA, ACh, NPY, SOM, and VIP on contractile amplitude and frequency in pigs partly differs between myometrium and endometrium/myometrium stripes [11,12,13,14,16,17,18,19]. Therefore, the role of GAL and its receptors was investigated using two kinds of uterine stripes. In all uteri used in the current experiment, NA decreased the contractility (amplitude, frequency), which confirmed the viability and usefulness of uterine tissues for research [16].

The completely new findings of the present experiment concern the participation of GAL in the contractility of the porcine uterus under physiological conditions and, for the first time, the role of this neuropeptide in the contractile activity of the uterus with inflammation. To date, the importance of GALR1 and GALR2 in the contractility of healthy and diseased uteri has not been examined. In the CON and SAL groups, this peptide reduced the amplitude and frequency in relation to the time before the GAL application. Only in one case was a statistically significant difference between both groups revealed, and it concerned the higher frequency in the endometrium/myometrium of the SAL group than in the CON group. As mentioned earlier, under physiological conditions, GAL stimulated the contractility of rat uteri [27,29] or did not significantly change this function in rats and perimenopausal-aged women [30,31]. It is known that GAL regulates gastrointestinal-tract motility, producing, for example, an inhibitory effect in guinea pig ileum [34] and stomach [46] and the opossum esophagus [47], as well as an excitatory effect in human [48] and rat [49] jejunum.

Compared to the period before GAL treatment, in the *E. coli* group, GAL reduced the amplitude in both types of stripes and the frequency in the endometrium/myometrium, and increased the value of the latter parameter in the myometrium. In these uteri, the frequency in the myometrium was increased compared to the CON and SAL groups, while in the endometrium/myometrium, this parameter was higher in relation to the CON group and was lower than in the SAL group. In turn, the amplitude in the inflamed uteri was similar to the values determined in the other groups. It is difficult to explain the varied GAL action on the frequency in both kinds of stripes of inflamed uteri. It may be because GAL increases the production and/or release in the myometrium of substances, enhancing contractility. It is known that GAL increases LTD_4_ synthesis in rat lungs [50], and that GAL and ACh act in a synergic way to induce the contraction of porcine [51] and goldfish [52] guts. Moreover, in the inflamed pig uteri, LTD_4_ increases the amplitude [14], while ACh the frequency [16].

Literature data show the role of GALR1 in the motility of guinea pig ileum [34,53] and the rat stomach [54], and GALR2 in the rat jejunum [49]. Considering the direction and level of significance of changes in response to GAL and GALR2 and GALR1/GALR2 antagonists, we indicated that these receptors also participate in the contractile activity of gilt uteri from the CON, SAL, and *E. coli* groups. In the healthy uterus, GAL reduced the amplitude mainly by GALR1, while use of the selective GALR2 antagonist allowed us to suppose that GAL, primarily by this receptor, decreased the frequency. In the inflamed uterus, GAL dropped the amplitude (in both kinds of stripes) and the frequency in endometrium/myometrium, and increased the frequency in the myometrium, mainly via GALR1. In the *E. coli* group, the alterations in the contractility in response to GAL were accompanied by a decreased myometrial GALR1 protein content and insignificant changes in the GALR2 protein level, as found in the present study. GAL could also regulate the contractility of healthy and inflamed uteri with GALR3 involvement, which was suggested regarding intestine motility [34]. The indirect GAL effect on uterine contractility connected with its action on the synthesis and/or secretion of substances participating in uterus contraction cannot be excluded. As mentioned above, GAL has the ability to increase LTD_4_ synthesis in the lungs [50], and together with ACh, to affect gut contraction [51,52]. The contractility effects of LTD_4_ [14] and ACh [16] were revealed earlier in healthy and inflammatory-changed pig uteri, as well as a marked increase in the LTs production in the pig uterus with inflammation [10]. However, the above suppositions require further research.

Regarding the clinical aspect of findings revealed in the present study, it can be hypothesized that by lowering the amplitude of the inflamed pig uterus, GAL favors the accumulation of inflammatory exudate inside the uterus, such as PGE_2_, NA, ACh, NPY, and VIP [11,13,16,17,19]. In turn, GAL increased myometrial frequency in the gilts of the *E. coli* group, similarly to PGF_2α_, PGI_2_, LTC_4_, and ACh [11,12,14,16]. Our data suggest that GAL, by increasing myometrial frequency in the inflamed uterus, contributes in the removal of inflammatory exudate from the uterine cavity to a limited extent. Moreover, the main participation of GALR1 in the GAL action on the contractile activity of inflammatory-changed uterus may be utilized for drug development to increase the contractility of the inflamed uterus. This may lead to an improvement in the effectiveness of treatment and the prevention of postpartum diseases of the reproductive system, and ultimately to better fertility rates and reduction of economic losses on farms.

## 4. Materials and Methods

### 4.1. Animals

The experimental procedures were performed in accordance with the relevant Polish and EU regulations in the field of Animal Protection and Welfare (Leg. Decree 26/2014 implementing EU directive 2010/63/EU), and were approved by the Local Ethics Committee (Consent no. 65/2015). The research was carried out on 15 gilts (Large White × Landrace, age 7–8 months, body weight (BW) 90–120 kg). A tester boar was used to determine behavioral estrus. In all gilts selected for the study, no disruptions in reproduction occurred: discharges from the vagina were not observed, and the second estrous cycle took place regularly. The animals were transported from a farm to the local animal house (University of Warmia and Mazury, Olsztyn, Poland) three days before the study. They were kept individually in pens (an area of about 5 m^2^) in the following conditions: temperature—18 ± 2 °C, natural daylight—14.5 ± 1.5 h, night—9.5 ± 1.5 h. The animals were fed with commercial diets and had access to water ad libitum.

### 4.2. Experimental Procedures

Following acclimatization, the gilts were allocated (randomly), on day 3 of the second estrous cycle (day 0 of the research), into the following groups of five animals in each group: *Escherichia coli* (*E. coli*) group, with intrauterine injections of *E. coli*; SAL group, with intrauterine injections of saline; and CON group, with a “sham” operation only.

The experimental procedures performed on animals have been described in detail previously [16]. The premedication was induced using atropine (0.05 mg/kg BW; Atropinum sulf. WZF, Warszawskie Zakłady Farmaceutyczne Polfa S.A., Poland), azaperone (2 mg/kg BW; Stresnil, Janssen Pharmaceutica, Beerse, Belgium), and ketamine hydrochloride (10 mg/kg BW; Ketamina, Biowet, Puławy, Poland). General anesthesia was induced with ketamine hydrochloride and maintained with supplementary doses (1 mg/kg BW every 5 min). Median laparotomy was then performed, and 50 mL of *E. coli* suspension (strain O25:K23/a/:H1; Department of Microbiology, National Veterinary Research Institute, Puławy, Poland) with 10^9^ colony-forming units/mL, was injected into each uterine horn in the *E. coli* group. In turn, in the SAL group, 50 mL of saline solution was injected. Bacterial suspension and saline were administered in five places (10 mL per injection) at a similar distance from each other. The uterine horns were then massaged to distribute bacterial suspension and saline evenly. In the animals of the CON group, only median laparotomy was done. All experimental gilts were left untreated in the period from surgery to euthanasia. On day 8 of the research (the expected day 11 of the estrous cycle), the pigs were euthanized with an overdose of ketamine hydrochloride, and the uteri were collected. For Western blotting, fragments of the horn were then collected from the following parts: paraoviductal, middle, paracervical. The uterine wall was divided into endometrial and myometrial layers using a scalpel blade and a dissecting microscope. The myometrium fragments (thickness of the entire layer) were snap-frozen in liquid nitrogen and stored at –80 °C for the Western blot method. For the immunofluorescent method, the horn fragments collected from three parts were divided into smaller pieces and placed in a 4% paraformaldehyde solution (pH 7.4) for 24 h. After fixation, the pieces were rinsed in 0.1 M phosphate-buffered saline (PBS, pH 7.4) and cryoprotected in an 18% buffered solution of sucrose (pH = 7.4) until sectioning. Fragments of the horn from its middle part were placed on ice and transported to the laboratory to study the uterine contractility (within 5 min following collection).

### 4.3. Western Blot Analysis

The myometrial tissues were homogenized on ice with a cold buffer containing: 50 mmol/L Tris-HCl, pH 8.0; 150 mmol/L NaCl; 1% Triton X-100, 10 mg/mL aprotinin, 52 mmol/L leupeptin, 1 mmol/L pepstatin A, 1 mmol/L EDTA, and 1 mol/L PMSF, and centrifuged (2500× *g*, at 4 °C, for 10 min). The supernatants were centrifuged (17,500× *g*, at 4 °C, for 1 h), and the collected supernatants were stored at −80 °C. The Bradford method was used to estimate the protein content [55]. Protein extracts (20 μg) were dissolved in a sodium dodecyl sulfate (SDS) gel-loading buffer (50 mmol/L Tris-HCl, pH 6.8; 4% SDS, 20% glycerol, and 2% β-mercaptoethanol), heated (95 °C, 4 min), and separated by 10% SDS-polyacrylamide gel electrophoresis. The separated proteins were then electro-blotted onto nitrocellulose membrane (0.22 μm) in a transfer buffer containing: 20 mmol/l Tris-HCl buffer, pH 8.2; 150 mmol/L glycine, 20% methanol, and 0.05% SDS. The membranes were incubated with 5% fat-free dry milk in a TBS-T buffer (at 21 °C, for 1.5 h) to block the nonspecific bindings. They were then incubated (at 4 °C, for 18 h) with primary antibodies: rabbit GALR1 polyclonal antibody (at a dilution of 1:500, cat. no. bs-9927R) and rabbit GALR2 polyclonal antibody (at a dilution of 1:500, cat. no. bs-11527R), both from Bioss Antibodies Inc. Following rinsing in TBS-T buffer, the membranes were incubated (at 21 °C, for 1 h) with biotinylated goat antirabbit IgG (at a dilution of 1:3000, cat. no. PK-6101, Vectastain Elite ABC-HRP Kit, Vector Labs, Burlingame, CA, USA). Incubation (for 3–4 min) with a mixture of 3, 30-diaminobenzidine tetrachloride (DAB, cat. no. D5637, Sigma Aldrich, St. Louis, MO, USA) and H_2_O_2_ in Tris-buffered saline (pH 7.2) was then conducted to visualize antibody binding. In order to demonstrate the specificity of the primary antibodies utilized, they were excluded from the analysis (negative control). Porcine duodenal protein was used as a positive control. Images were gained and quantified by using a Quantity-One system (VersaDoc 4000M imaging system, Bio-Rad Laboratories, Hercules, CA, USA). The density of bands was normalized in relation to the protein content of glyceraldehyde-3-phosphate dehydrogenase (GAPDH).

### 4.4. Immunofluorescence

The pieces of uterine horns were cut in a cryostat (Reichert-Jung, Nußloch, Germany), and the obtained sections (10 μm thickness) were stained using the single-immunofluorescent method, as previously described [17]. Briefly, after drying (at 21 °C, for 30 min) and rinsing (0.1M PBS, pH = 7.4, three times, each for 15 min), uterine sections were incubated (at 21 °C, for 1 h) with a buffered blocking mixture with the following composition: 0.1 M PBS, 10% normal goat serum (MP Biomedicals, Solon, OH, USA), 0.1% bovine serum albumin (Sigma-Aldrich, St. Louis, MO, USA), 0.05% Thimerosal (Sigma-Aldrich, St. Louis, MO, USA), 1% Triton X-100 (Sigma-Aldrich, St. Louis, MO, USA), and 0.01% sodium azide. Following subsequent washing (as given above), the tissue sections were incubated (at 21 °C, for 18 h) in a humidity chamber, with primary antibodies. The same antibodies as for the Western blot method were used: against GALR1 (at a dilution of 1:100) and GALR2 (at a dilution of 1:2500). On the next day, the sections were washed (as given above) and incubated with biotinylated antirabbit IgG (at a dilution of 1:1000, cat. no. AP132B, Chemicon International, Temecula, CA, USA) (at 21 °C, for 1 h), and then with carbocyanine 3 (CY3)-conjugated streptavidin (at a dilution of 1:9000, cat. no. 016160084, Jackson ImmunoResearch Labs, West Grove, PA, USA) (at 21 °C, for 1 h). Rabbit normal IgG was used instead of primary antibodies to perform the negative control. As a positive control, sections of the porcine duodenum were used. Immunoreactivity was assessed using the microscope with epi-fluorescence and appropriate filters (Olympus BX51, Olympus Consilio sp. z.o.o., Warsaw, Poland).

### 4.5. Preparation of the Stripes from the Uteri and Measurement of Isometric Contractile Function

In the present study, to carry out the recording of contractile activity of the uteri, two types of stripes were also collected from each organ [13]. The stripes (approximate size 3 × 5 mm) of myometrium and endometrium/myometrium were rinsed in saline and mounted between two stainless steel hooks in an organ bath with a capacity of 10 mL (Radnoti Unit Tissue Organ Bath System type 159920, Germany) under 5 mN tension. A Krebs–Ringer solution containing (mM/L): NaCl, 120.3; KCl, 5.9; CaCl_2_, 2.5; MCl_2_, 1.2; NaHCO_3_, 15.5; glucose, 11.5; and pH 7.4, was placed in the organ bath. The Krebs–Ringer solution was at a temperature of 37 °C, and was constantly saturated with a mixture of 95% O_2_ and 5% CO_2_.

### 4.6. Contractility Study Schedule

**Figure 6:**
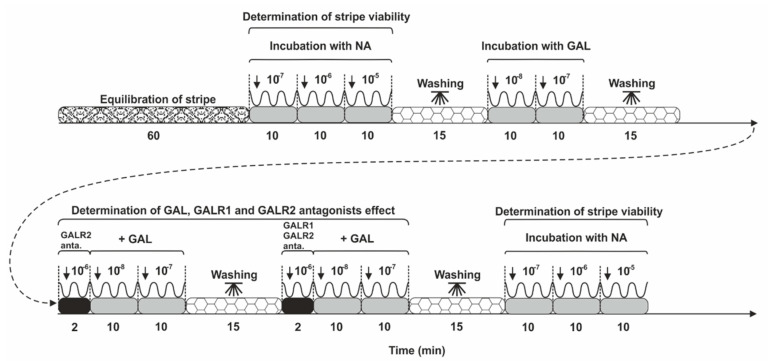
Scheme of the management of stripes of the uterus. NA—noradrenaline, GAL—galanin, GALR2 anta.—GAL receptor subtype 2 antagonist, GALR1/GALR2 anta.—GAL receptor subtypes 1 and 2 antagonist. The concentrations of these factors are given in moles.

The procedure for handling uterine stripes is shown in the scheme in Figure 6. Following equilibration, the spontaneous contractility of the uterine stripes was registered (for 1 h). Amplitude (the difference between the minimum and maximum values for a single contraction in mN) and frequency (the number of peaks) of the stripes were measured with a force-displacement transducer and analyzed with a computer using PowerChart software (Chart v5, scope v5) from AD Instruments. First, to determine the viability of uterine tissues and their usefulness for further research, stripes were treated with NA (Levonor, Warszawskie Zakłady Farmaceutyczne Polfa, Poland) at the following doses: 10^−7^, 10^−6^, or 10^−5^ M. The findings of NA action have been reported previously [16]. Next, the effect of GAL (doses: 10^−8^, 10^−7^ M, cat. no. H-1365.0500, Bachem) on the uterine contractility was determined. The influence of each dose of GAL was registered for 10 min. Uterine contractility was also investigated in response to GAL in the presence of GALR2 antagonist (M871, Ki values: 13.1 and 420 nM for GALR2 and GALR1, respectively, cat. no. AB141159) and GALR1/GAL2R antagonist (M40, Ki values: 1.82 and 5.1 nM at GALR1 and GALR2, respectively, cat. no. AB141157) both from Abcam. For this, the uterine stripes were first treated with the antagonists (GALR2 or GALR1/GALR2, each at a dose of 10^−6^ M, for 2 min), and GAL (doses: 10^−8^, 10^−7^ M) was then administered. The effects of antagonists and GAL were measured for 10 min. Each measurement was finished with the rinsing of stripes (in PBS, three times). At the end of the investigation, the viability of tissues was checked again by NA administration at the doses given above. Only findings registered from tissues in which the discrepancies in reaction to NA at the start and end of the research that were under 20% were considered. The NA doses were used in previous experiments [12,13], while GAL and antagonist concentrations were selected in the initial experiment. Using healthy porcine uteri and GAL (doses: 10^−9^, 10^−8^, 10^−7^ M) alone and together with particular antagonists (doses: 10^−8^, 10^−7^, 10^−6^ M), it was found that GAL at doses of 10^−8^ and 10^−7^ M more effectively decreased the contractile parameters, while antagonists at a dose of 10^−6^ M resulted in statistically significant changes in the GAL-influenced contractile parameters (data not shown).

### 4.7. Statistical Analyses

The mean (±SEM) protein expression of GALR1 and GALR2 was counted for each studied group, and then the statistical significances between obtained data were evaluated by the Bonferroni test (ANOVA, InStat Graph Pad, San Diego, CA, USA). The mean (±SEM) values of amplitude and frequency counted for each group before adding substances (pretreatment period) were accepted as 100%. The influences of substances were expressed as percentage (mean ± SEM) values of these parameters measured before their use. Comparisons were performed using the Bonferroni test to analyze the contractile function between the mean values before and following each treatment in each group, and the mean values between groups in response to the same treatment. The following thresholds were used to mark statistically significant differences: * *p* < 0.05, ** *p* < 0.01, *** *p* < 0.001.

## 5. Conclusions

The current study demonstrated that the inflammatory state reduces GALR1 protein expression in the porcine myometrium. The study also showed that in the inflamed uterus, GAL, acting mainly through GALR1, reduces the contractile amplitude in the myometrium and endometrium/myometrium and the frequency in the endometrium/myometrium, and increases the frequency in the myometrium. Thus, the obtained results allowed us to suppose that GAL may participate in altered uterine contractility in the course of spontaneous inflammation. Moreover, the present study should prompt research into the mechanisms underlying changes in GALRs expression with a view toward their possible therapeutic role in increasing the myometrial contractility of an inflamed uterus.

## Data Availability

All relevant data are within the manuscript.

## Supplementary Materials

The following are available online at https://www.mdpi.com/article/10.3390/ijms22126415/s1, Figure S1: Western blotting of GALR1 and GALR2 in porcine myometrium and relevant positive control, Figure S2: The immunoexpression of GALR1 and GALR2 in porcine myometrium and relevant positive control.
