# Supplementary material for: Regulatory Influence of Galanin and GALR1/GALR2 Receptors on Inflamed Uterus Contractility in Pigs
Source: Int J Mol Sci. 2021 Jun 15;22(12):6415. doi: 10.3390/ijms22126415 (PMC8232690; doi:10.3390/ijms22126415)
Supplement: Supplementary file 1 [file ijms-22-06415-s001.zip › ijms-1248122-supplementary.pdf]

Supplementary Materials

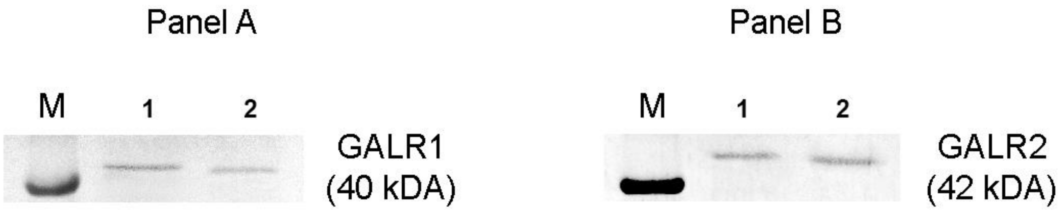

Figure S1. Western blotting of GALR1 and GALR2 in porcine myometrium and relevant positive control. Panel A shows blots for GALR1 antibody: specific bands at 40 kDa are visible in porcine myometrium (line 1) and duodenum (line 2). Panel B shows blots for GALR2 antibody: specific bands at 42 kDa are visible in porcine myometrium (line 1) and duodenum (line 2). M - marker.

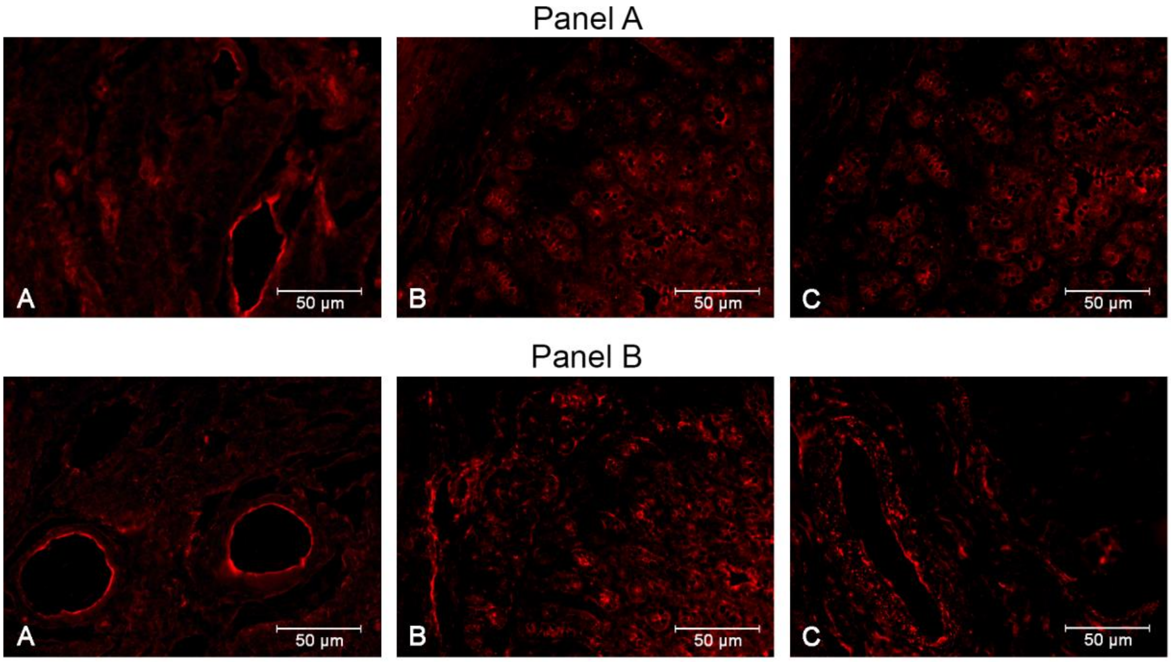

Figure S2. The immunoexpression of GALR1 and GALR2 in porcine myometrium and relevant positive control. Panel A shows the presence of GALR1 in porcine myometrium (**A**) and duodenum (**B**, **C**). Panel B shows the presence of GALR2 in porcine myometrium (**A**) and duodenum (**B**, **C**).
